# Evaluating heart electrical activities and cardiac arrhythmias of Holstein cows during ageing by short‐term electrocardiography in comparison with 24‐hour holter‐monitoring

**DOI:** 10.1002/vms3.448

**Published:** 2021-02-15

**Authors:** Aliasghar Chalmeh, Sanaz Karamifar

**Affiliations:** ^1^ Department of Clinical Sciences School of Veterinary Medicine Shiraz University Shiraz Iran

**Keywords:** ageing, cardiac arrhythmia, cow, electrocardiography, herat electrical activity, Holter‐monitoring

## Abstract

Short‐term electrocardiography is one of the most suitable tools to study the electrical activity of the heart, but the use of a tool such as a Holter‐monitor with the ability to assess the long‐term of the heart electrical activity, can provide more accurate information about these activities by comparing the results. It is possible to understand the superiority of each over the other and the resulting differences. Therefore, 60 female Holstein cows in 10 age groups, including 1 day, 1, 3, 6 months, 1, 2, 3, 4, 6 and 8 years were included in the study (6 heads in each age group). Electrocardiography (for 5 min) and Holter‐monitoring (for 24 hr) were performed from the entire study population. The Q, R and T amplitudes in electrocardiography were significantly higher than those in Holter‐monitoring. The P, R and T durations and P‐R, R‐R, Q‐T and S‐T intervals at all ages were significantly longer in the Holter‐monitoring than in the electrocardiographic method. The heart rate of animals was significantly lower in the 24‐hr Holter‐monitoring than in the short‐term electrocardiography. The trend of changes of all parameters was significant during ageing. Cardiac arrhythmias included sinus arrhythmia and sino‐atrial block, which were the most common cardiac arrhythmias in the 24‐hr Holter‐monitoring. It appears that long‐term Holter‐monitoring is a more reliable method than short‐term electrocardiography to assess cardiac arrhythmias. Additionally, the indicators of electrical activity of the heart (waves) in the Holter‐monitoring method are significantly different from short‐term electrocardiography, which is probably due to the collection of information over a long period and in non‐stressful situations. Furthermore, it appears that the use of the 24‐hr Holter‐monitoring method is preferable to the short‐term electrocardiography method to evaluate the electrical activity of the heart of cows at all ages.

## Graphical abstract

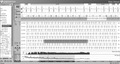

## 1 INTRODUCTION

The cardiovascular system is vital in the body that is responsible for supplying blood to various organs. Cardiac activities include electrical and mechanical functions, and the proper functioning of the heart's mechanical activity depends on the accuracy of its electrical performance (Constable et al., 2017). Considering the importance of the heart's electrical function, the use of methods to assess this activity is clinically essential. Electrocardiography (ECG) is an inexpensive and non‐invasive technique to evaluate heart electrical activity that provides useful information to classify arrhythmias and diagnose conduction abnormalities of the heart, as well as helps to predict the patient and evaluate treatment processes. The physiological and anatomical features of the heart in large animals, including cattle, are different from other farm animals. Therefore, the base‐apex lead has been suggested for bovine electrocardiography (Constable et al., 2017). The base‐apex lead is easy to handle and animal movements have the least negative effects on the recorded waves (Rezakhani et al., 2010).

Short‐term ECG is one of the most suitable tools to study the electrical activity of the heart, but alongside the benefits of this technique, there are some disadvantages, including lack of detection of the heart's electrical problems during short‐term recordings. In addition, this method is unsuitable for the long‐term monitoring of patients’ hearts. Furthermore, long‐term attachment of electrocardiograph clips to the skin may cause pressure and stimulate the sensation of pain, causing problems in continuous and prolonged ECG recordings (Mazrouei Sebdani et al., 2019). Hence, the use of a method to reduce the disadvantages of the standard ECG was necessary. Accordingly, the use of Holter‐monitor in modern cardiology has been suggested as a stress‐free technique to evaluate long‐term cardiac electrical activities (Kuwahara et al., 2004).

Holter‐monitoring is an efficient method to evaluate long‐term cardiac electrical activities by using electrodes attached to the chest. The number and location of these electrodes vary depending on the type and model of the device, but most of them have between 3 and 8 electrodes. This device is portable and contains hardware (to view, control and record signals) and software (to review and process the recorded information) (Hiraga & Sugano, 2015). It appears that Holter‐monitoring with the ability to assess the long‐term cardiac electrical activity can provide more accurate information about these activities by comparing the results to short‐term ECG.

Physiological and anatomical changes occurring in the cardiovascular system can also affect the cardiac electrical activity. Physiological changes, such as growth, pregnancy, parturition and lactation, cause changes in the electrical activity of the heart. Numerous studies on behavioural, physiological and immunological changes in cows under different conditions and ages have been performed, but there are no comprehensive studies on changes in the cardiac electrical activity of cows during ageing. According to the mentioned factors, it appears that ageing is one of factors affecting the cardiac electrical activity.

According to advantages and disadvantages of ECG and Holter‐monitoring as well as the necessity and importance of cardiac electrical activity monitoring during ageing, both techniques were used at different ages of Holstein cows in this study. Subsequently, comparison of the parameters recorded by each of them may indicate their reliability in assessing the cardiac electrical performance of the cows.

## 2 MATERIALS AND METHODS

### 2.1 Animals

Referring to an industrial dairy farm around Shiraz, southwest of Iran (latitude of 29° 32' N and longitude 52°35′ E, 1,810 m above sea level), in October 2020, 60 Holstein cows in 10 equal age groups, including 1 day, 1, 3 and 6 months, 1, 2, 3, 4, 6 and 8 years, were selected. Animals were kept in an open‐shed system with free access to water and shade, and balanced rations based on the NRC requirements were provided. All animals were clinically evaluated before the start of the study, and their clinical health was confirmed, especially from the perspective of cardiovascular health. In addition, the animals had no history of severe debilitating diseases. At the beginning of the study, at 09:00 a.m., ECG was taken from the entire study population for 5 min as described below, and then by installing Holter‐monitor, their heart electrical activities were continuously evaluated for 24 hr.

### 2.2 Electrocardiography

Initially, electrocardiograms were taken for 5 min from all of the studied animals with a base‐apex lead system (25 mm/s; 10 mm/mV; Kenzline EKG 110, Suzuken Co., Ltd.). Before attaching the electrodes to the skin, the sites were prepared with alcohol and an electrocardiogram jelly. In all animals, the positive electrode (left arm) was attached to the apex of the heart between the fifth or sixth intercostal space along the left olecranon, the negative electrode (right arm) on the jugular furrow at the top of the heart base, and the neutral electrode was placed along the spine at a location far from the heart (Constable et al., 2017).

### 2.3 Holter‐monitoring

After recording the electrocardiograms, the animals were at rest for 30 min, then 4 points of their body were washed with alcohol, and the electrodes of the Holter‐monitor (Cardio Rhythm, ACT^®^ Eng. Co. Ltd.) were attached for a continuous 24‐hr recording period (25 mm/s; 10 mm/mV). A positive electrode was attached to the apex of the heart between the fifth and sixth intercostal space near the left olecranon, another positive electrode was attached to the jugular furrow at the top of the heart base, the negative electrode was attached to the left side of the chest near the spine, and the earth electrode was placed in the lower left half of the chest, aligned with the negative electrode (Mazrouei Sebdani et al., 2019). All electrodes were attached to the skin by lead lock ECG electrodes, purchased from Medico Electrodes Company, India. After 24 hr, the Holter‐monitor was separated from the animals, and the data recorded by the software were analysed.

### 2.4 Evaluation of the recorded samples

After recording the cardiac electrical activities by both devices, different indices including durations, amplitudes, intervals, heart rates and arrhythmias, were evaluated.

### 2.5 Statistical analyses

The normality of the data was evaluated based on the results of the Kolmogorov–Smirnov test. The results of this test indicated that all data were normally distributed. The data were presented as the mean ± standard error of mean (*SEM*). Comparison of the mean of similar parameters recorded by the two different techniques at each age group was performed by two independent samples *t*‐test. The trend changes of each parameter by recorded electrocardiogram (within 5 min) and Holter‐monitor (during 24 hr) were evaluated by the Repeated Measures ANOVA. All statistical analyses were conducted by the SPSS software, version 22, and the level of significance was considered at *p* < .05.

## 3 RESULTS

**FIGURE 1:**
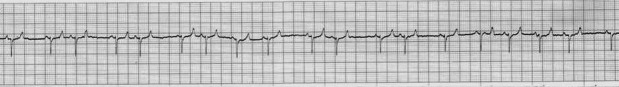
An electrocardiogram recorded from a 3‐month‐old female Holstein calf with sinus arrhythmia (Base‐apex lead electrocardiography; 25 mm/s; 10 mm/mV)

**FIGURE 2:**
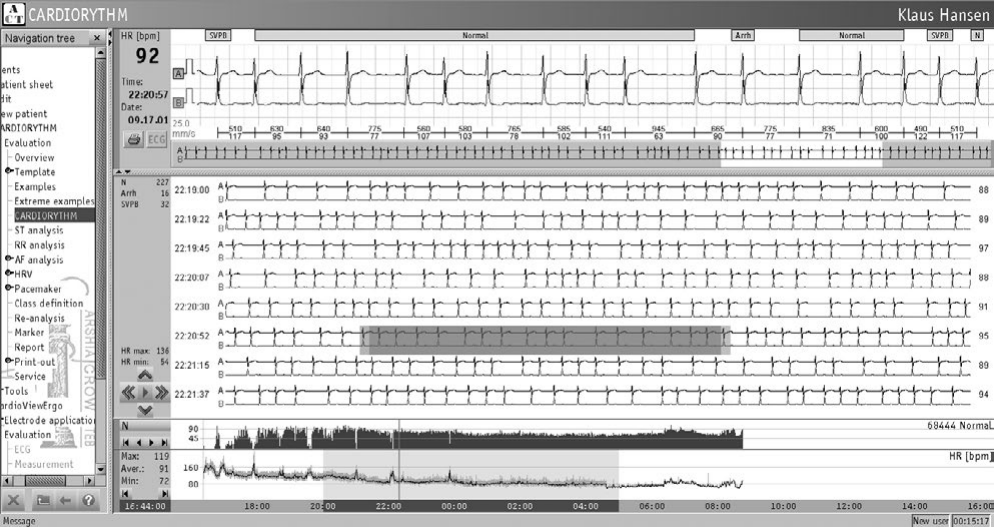
Recorded heart electrical activity of a 6‐month‐old female Holstein calf by 24‐hr Holter‐monitoring (25 mm/s; 10 mm/mV). Sinus arrhythmia and sino‐atrial block arrhythmia are present

**FIGURE 3:**
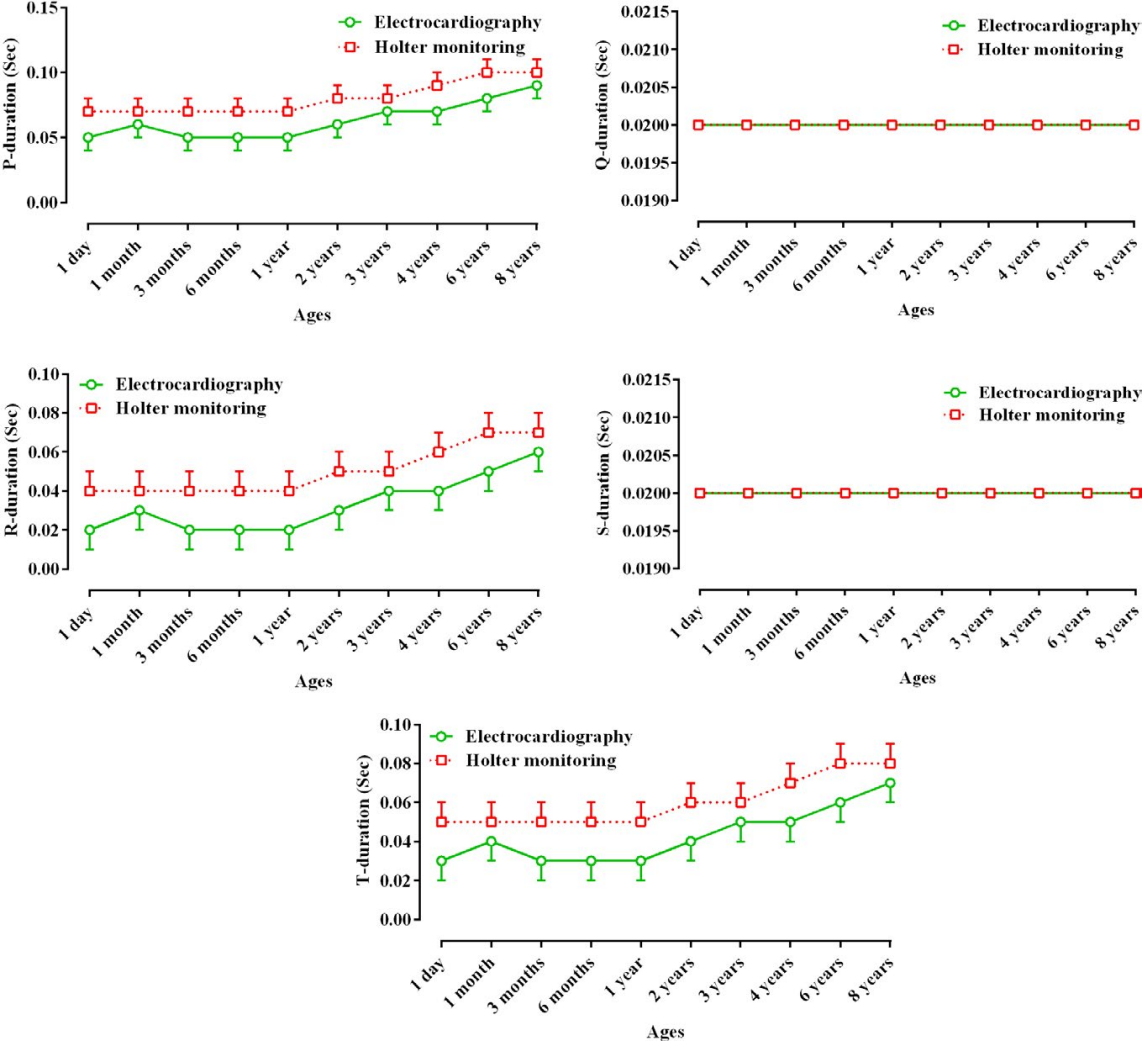
Comparative trend changes of P, Q, R, S and T durations (Mean ± *SD*) during ageing in Holstein cows recorded by short‐term electrocardiography and 24‐hr Holter‐monitoring

**FIGURE 4:**
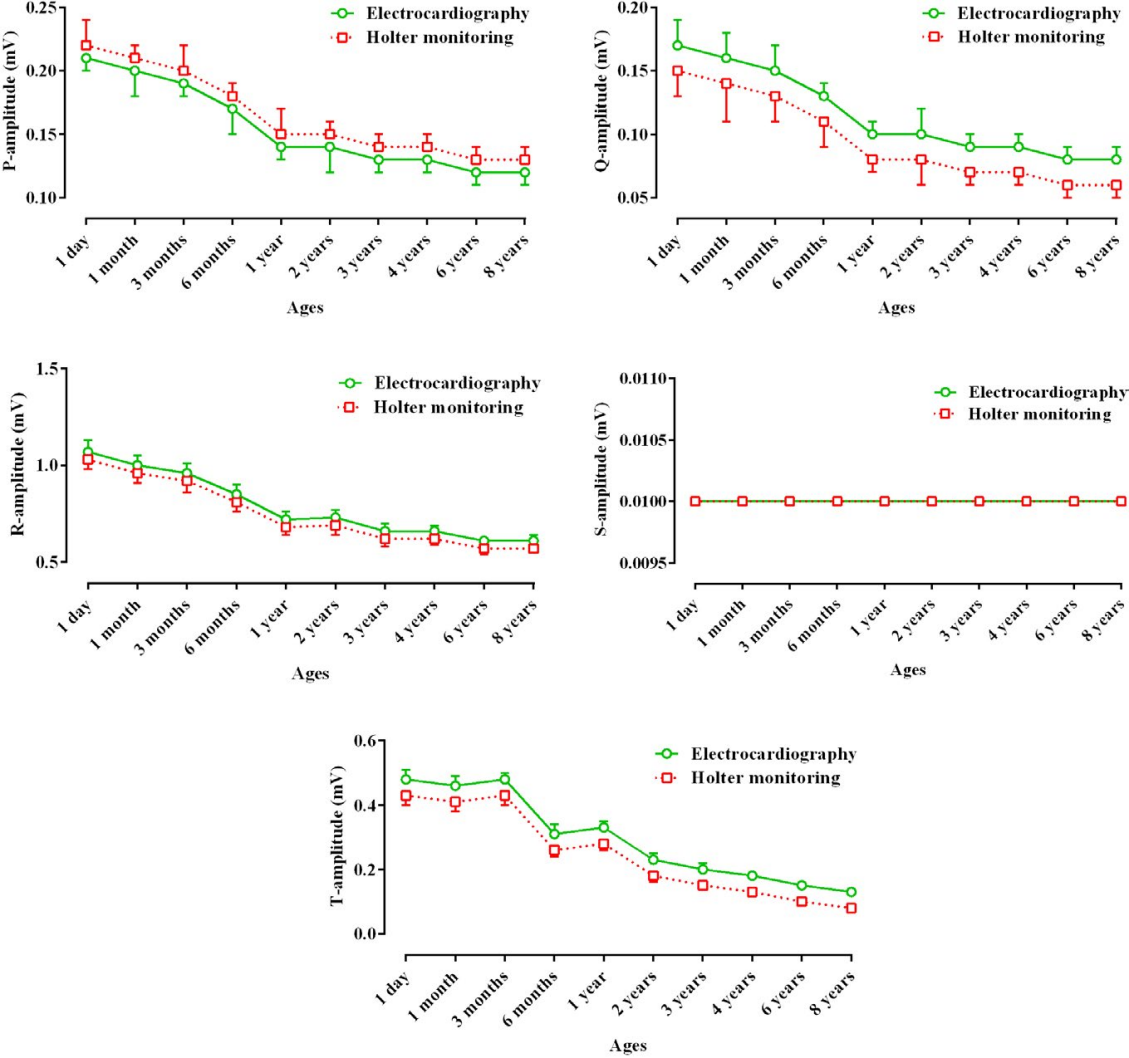
Comparative trend changes of P, Q, R, S and T amplitudes (Mean ± *SD*) during ageing in Holstein cows recorded by short‐term electrocardiography and 24‐hr Holter‐monitoring

**FIGURE 5:**
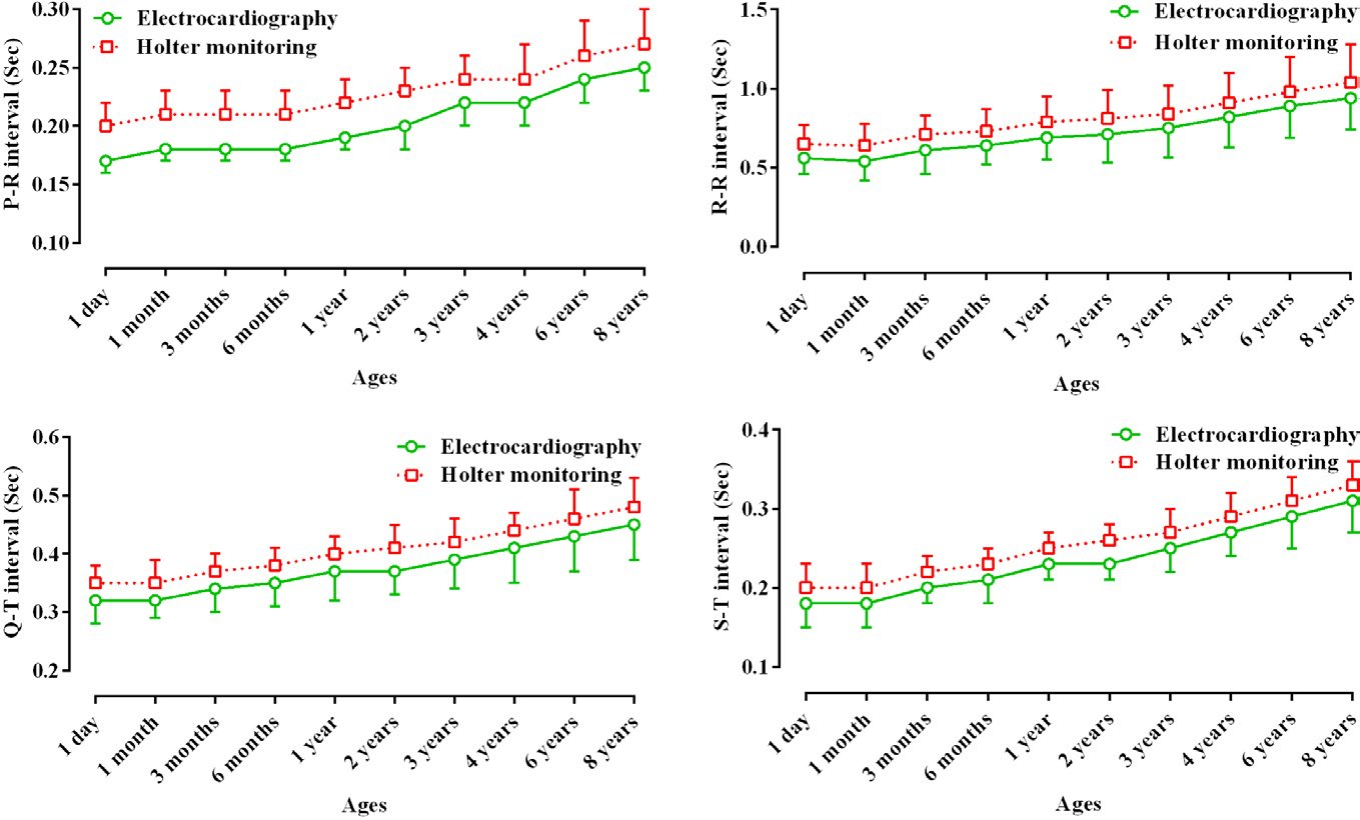
Comparative trend changes of P‐R, R‐R, Q‐T and S‐T intervals (Mean ± *SD*) during aging in Holstein cows recorded by short‐term electrocardiography and 24‐hr Holter‐monitoring

**FIGURE 6:**
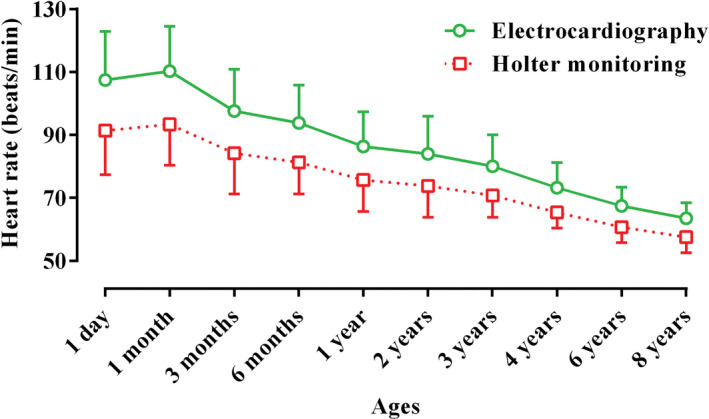
Comparative trend changes of heart rate (Mean ± *SD*) during ageing in Holstein cows recorded by short‐term electrocardiography and 24‐hr Holter‐monitoring

Table 1 presents the recorded parameters by short‐term ECG and 24‐hr Holter‐monitoring at different ages of Holstein cows. Statistically, for some parameters (P and S amplitudes, and Q and S durations), there was no difference between the two techniques, and the trend of changes in these waves from 1 day to 8 years was not significant (*p* < .05; Table 1; Figures 3 and 4). The Q, R and T amplitudes in ECG were significantly higher than those in Holter‐monitoring, and the trend of changes in these indices during ageing was significant (*p* < .05; Table 1; Figure 4). The P, R and T durations at all ages were significantly longer in Holter‐monitoring than in ECG, and the trend of changes in these parameters was significant (*p* < .05; Table 1; Figure 3). The P‐R, R‐R, Q‐T and S‐T intervals at all ages were significantly longer in Holter‐monitoring than in ECG, and the trend of changes in these intervals during ageing was significant (*p* < .05; Table 1; Figure 5). The heart rate of the animals in the 24‐hr Holter‐monitoring was significantly lower than that in the short‐term ECG. The trend of changes in the heart rate during ageing was decreasing and significant (*p* < .05; Table 1; Figure 6). According to Table 2, some animals had normal sinus rhythm, and the arrhythmias detected in others included sinus arrhythmias and sino‐atrial block (Figures 1 and 2). The highest frequency of cardiac arrhythmias was in the 24‐hr Holter‐monitoring technique (Table 2).

**TABLE 1:** Cardiac electrical activities by short‐term electrocardiography compared to 24‐hr Holter‐monitoring in Holstein cows during ageing (Mean ± *SEM*)

Parameters	Techniques	Ages										*SEM*	*P*‐value
		1 day	1 month	3 months	6 months	1 year	2 years	3 years	4 years	6 years	8 years		
P‐amplitude (mV)	Electrocardiography	0.21	0.20	0.19	0.17	0.14	0.14	0.13	0.13	0.12	0.12	0.004	<.001
	Holter monitoring	0.22	0.21	0.20	0.18	0.15	0.15	0.14	0.14	0.13	0.13	0.004	<.001
Q‐amplitude (mV)	Electrocardiography	0.17a	0.16a	0.15a	0.13a	0.10a	0.10a	0.09a	0.09a	0.08a	0.08a	0.004	<.001
	Holter monitoring	0.15b	0.14b	0.13b	0.11b	0.08b	0.08b	0.07b	0.07b	0.06b	0.06b	0.004	<.001
R‐amplitude (mV)	Electrocardiography	1.07a	1.00a	0.96a	0.85a	0.72a	0.73a	0.66a	0.66a	0.61a	0.61a	0.021	<.001
	Holter monitoring	1.03b	0.96b	0.92b	0.81b	0.68b	0.69b	0.62b	0.62b	0.57b	0.57b	0.021	<.001
S‐amplitude (mV)	Electrocardiography	0.01	0.01	0.01	0.01	0.01	0.01	0.01	0.01	0.01	0.01	0.001	NS
	Holter monitoring	0.01	0.01	0.01	0.01	0.01	0.01	0.01	0.01	0.01	0.01	0.001	NS
T‐amplitude (mV)	Electrocardiography	0.48a	0.46a	0.48a	0.31a	0.33a	0.23a	0.20a	0.18a	0.15a	0.13a	0.018	<.001
	Holter monitoring	0.43b	0.41b	0.43b	0.26b	0.28b	0.18b	0.15b	0.13b	0.10b	0.08b	0.018	<.001
P‐duration (s)	Electrocardiography	0.05a	0.06a	0.05a	0.05a	0.05a	0.06a	0.07a	0.07a	0.08a	0.09a	0.001	<.001
	Holter monitoring	0.07b	0.07b	0.07b	0.07b	0.07b	0.08b	0.08b	0.09b	0.10b	0.10b	0.001	<.001
Q‐duration (s)	Electrocardiography	0.02	0.02	0.02	0.02	0.02	0.02	0.02	0.02	0.02	0.02	0.001	NS
	Holter monitoring	0.02	0.02	0.02	0.02	0.02	0.02	0.02	0.02	0.02	0.02	0.001	NS
R‐duration (s)	Electrocardiography	0.02a	0.03a	0.02a	0.02a	0.02a	0.03a	0.04a	0.04a	0.05a	0.06a	0.001	<.001
	Holter monitoring	0.04b	0.04a	0.04b	0.04b	0.04b	0.05b	0.05a	0.06b	0.07b	0.07a	0.001	<.001
S‐duration (s)	Electrocardiography	0.02	0.02	0.02	0.02	0.02	0.02	0.02	0.02	0.02	0.02	0.001	NS
	Holter monitoring	0.02	0.02	0.02	0.02	0.02	0.02	0.02	0.02	0.02	0.02	0.001	NS
T‐duration (s)	Electrocardiography	0.03a	0.04a	0.03a	0.03a	0.03a	0.04a	0.05a	0.05a	0.06a	0.07a	0.001	<.001
	Holter monitoring	0.05b	0.05a	0.05b	0.05b	0.05b	0.06b	0.06a	0.07b	0.08b	0.08a	0.001	<.001
PR‐interval (s)	Electrocardiography	0.17a	0.18a	0.18a	0.18a	0.19a	0.20a	0.22a	0.22a	0.24a	0.25a	0.003	<.001
	Holter monitoring	0.20b	0.21b	0.21b	0.21b	0.22b	0.23b	0.24b	0.24b	0.26b	0.27b	0.003	<.001
RR‐interval (s)	Electrocardiography	0.56a	0.54a	0.61a	0.64a	0.69a	0.71a	0.75a	0.82a	0.89a	0.94a	0.005	<.001
	Holter monitoring	0.65b	0.64b	0.71b	0.73b	0.79b	0.81b	0.84b	0.91b	0.98b	1.04b	0.005	<.001
QT‐interval (s)	Electrocardiography	0.32a	0.32a	0.34a	0.35a	0.37a	0.37a	0.39a	0.41a	0.43a	0.45a	0.005	<.001
	Holter monitoring	0.35b	0.35b	0.37b	0.38b	0.40b	0.41b	0.42b	0.44b	0.46b	0.48b	0.005	<.001
ST‐interval (s)	Electrocardiography	0.18a	0.18a	0.20a	0.21a	0.23a	0.23a	0.25a	0.27a	0.29a	0.31a	0.016	<.001
	Holter monitoring	0.20b	0.20b	0.22b	0.23b	0.25b	0.26b	0.27b	0.29b	0.31b	0.33b	0.016	<.001
HR (beats/min)	Electrocardiography	107.42a	110.25a	97.61a	93.79a	86.35a	83.93a	80.00a	73.19a	67.43a	63.53a	2.001	<.001
	Holter monitoring	91.35b	93.40b	84.18b	81.33b	75.67b	73.81b	70.75b	65.37b	60.74b	57.55b	1.532	<.001

Note

Different letters indicate significant difference between two techniques for each parameter in each age group (*p* < .05). The trend of changes in each parameter for each of the assessment methods during ageing is shown in the right column (*p* < .05).

**TABLE 2:** Frequency and distribution of cardiac arrhythmias recorded by short‐term electrocardiography and 24‐hr Holter‐monitoring in each age group

Cardiac Arrhythmias	Techniques	Ages									
		1 day	1 month	3 months	6 months	1 year	2 years	3 years	4 years	6 years	8 years
NSR	Electrocardiography	1	1	1	2	2	3	2	3	4	3
	Holter monitoring	0	0	0	0	0	0	0	0	0	0
SA	Electrocardiography	5	5	5	3	4	2	3	3	1	2
	Holter monitoring	6	6	6	5	6	5	5	6	5	5
SAB	Electrocardiography	0	0	0	1	0	1	1	0	1	0
	Holter monitoring	0	0	0	1	0	1	1	0	1	1

Abbreviations: NSR, normal sinus rhythm; SA, sinus arrhythmia; SAB, sino‐atrial block.

## 4 DISCUSSION

There are several methods to assess the cardiovascular system in cattle such as peripheral pulse, mucous membrane colour, capillary refill time, apex beat palpation, jugular pulses, auscultation of heart sounds, cardiac percussion, ultrasonography, radiography, pericardiocentesis, microbial blood cultures, complete blood counts, circulating electrolytes and cardiovascular biomarkers, ECG, and Holter‐monitoring (Constable et al., 2017).

Evaluation of cardiac electrical activity is clinically necessary to confirm the health of the animal, and the use of an effective, efficient and accurate method is required. ECG is one of the methods defined for this purpose, but in some cases, some heart disorders may not occur in the short time that the device is attached to the animal, and therefore the heart electrical activity should be monitored for a longer period of time. Accordingly, this study evaluated the heart electrical activity of Holstein cows by the standard short‐term ECG and 24‐hr Holter‐monitoring, and then compared the information obtained from these two techniques to determine which method was more effective and efficient in assessing the electrical activity of the heart.

Based on the data obtained from this study, most of the parameters recorded by 24‐hr Holter‐monitoring were significantly different from the short‐term ECG (Table 1). It may be suggested that the circadian physiological changes in the animal's body during this study may be considered a reason for these differences. Continuous 24‐hr study of cardiac electrical activity encompasses high and low conduction periods during the course of the day, and the data presented in Table 1 are the average of a large volume of data recorded by Holter‐monitoring. Based on the non‐stressful conditions of the studied animals and the continuality of 24‐hr evaluations, it may be suggested that data from Holter‐monitoring for all the recorded parameters are more reliable than the data from the short‐term ECG. In other words, evaluation of heart electrical activity by the short‐term ECG may cause stressful conditions for the cows, while 24‐hr Holter‐monitoring does not cause this amount of stress to the animals and causes differences in the results obtained.

Cardiac rhythm evaluation in the studied cows indicated that the distribution and variability of heart rhythm were different in both techniques. In some of the studied cows by other researchers, the atrio‐ventricular block was identified only by Holter‐monitoring, which may be due to increased tonicity of the vagus nerve at rest (Guccione et al., 2014). In the present study, sinus arrhythmia was detected by both methods in all studied cows (Table 2). Researchers have reported that sinus arrhythmia is the most physiological arrhythmia in large animals (Hesselkilde et al., 2014; Constable et al., 2017), and in this study, we obtained the same finding.

In the current research, the frequency of cardiac arrhythmias was higher in Holter‐monitoring than in ECG, which is probably due to the increased vagus nerve tonicity during rest and non‐stress times. Pessoa et al. (2016) examined the electrical activity of the heart of 10 male Holstein calves (3–6 months old) by 24‐hr Holter‐monitoring. They assessed only the number of heart beats per minute as well as the presence of cardiac arrhythmias, and in addition to normal sinus rhythm, they identified arrhythmias such as sinus arrhythmia, second degree atrioventricular block and supraventricular premature beat. In another study, Frese et al. (2017) suggested that 24‐hr Holter‐monitoring could be used to examine the cardiac arrhythmias of cows. In some older studies, conventional ECG with longer periods of time examined the cardiac electrical activity of cows in different conditions, but in these studies, the usual methods of Holter‐monitoring were not used (Mohr et al., 2002; Hagen et al., 2005; Gyrax et al., 2008; Schmied et al., 2008).

Very limited researches have been conducted on the long‐term evaluation of the electrical activity of cows' hearts using Holter‐monitoring, and none of them had a comprehensive comparison between short‐term ECG and 24‐hr Holter‐monitoring during ageing. Scheer et al. (2005) have suggested that small ruminants like sheep are an ideal animal for Holter‐monitoring, since they are big enough, are quiet and have non‐destructive behaviour. Furthermore, Kviesulaitis et al. (2018) stated that sheep could be an excellent research subject for large animal experimental studies owing to their heart structure and physiology, which have considerable similarity to those of humans.

Figures 3, 4, 5, 6 present alterations of cardiac electrical indices during ageing. As these figures and Table 1 present, almost all indices changed during ageing. Some parameters increased and some of them decreased. With advancing age, degenerative changes occur in the heart muscle and its conduction system. Some of the pathways of the pacemaker system may develop fibrous tissues and fat deposits (O'Connor et al., 2008). Furthermore, many of the changes occurring in the electrocardiogram reflect the anatomical dominance of the right ventricle during early months of life. At birth, the right ventricle is thick owing to high pulmonary artery pressure *in utero*. With the expected fall in the pulmonary artery pressure during the neonatal period, the right ventricular wall stress and thickness decrease until the right ventricular pressure leads to approximate adult amounts (Bansal & Bansal, 2012).

In the current study, amplitudes were higher in younger animals than in adult ones (Figure 4). It may be suggested that the thinner chest wall in young animals allows the fast transmission of cardiac electrical waves to recording devices. The conductive properties of the body mass of ruminants, attributable to the volume of the gastrointestinal tract, influence the distribution of the body surface potential comprising the electrocardiogram (Santamarina et al., 2001). It is possible that gradual development of body mass may cause difficulty in reaching the waves to the body surface due to relative electrical insulation by increasing body mass and changing in amplitudes can occur. It can be suggested that as the mass of the heart in larger animals becomes larger in the process of growth, the duration of transfer of the cardiac electrical activity also increases. Furthermore, ruminants have a deeply penetrating purkinje system, and depolarization from ventricular endocardium to epicardium occurs explosively and in many directions at once (Pennisi et al., 2002).

Our study indicated that the values of durations (Figure 3) and intervals (Figure 5) were significantly higher in older animals than in younger ones (*p* < .05; Table 1), which might be due to the larger size of the heart in older cows. With advancing age, widespread histological changes in the conduction system occur. These changes may alter several features of the ageing electrocardiogram, orientation of the electrical axis, duration and morphology of the atrial and ventricular complexes, and characteristics of the ventricular repolarization (Chalmeh et al., 2013).

## 5 CONCLUSION

In conclusion, cardiac electrical activities of cows were altered during ageing, and theses alterations were related to the physiological changes of heart. Furthermore, recorded indices of cardiac electrical functions by the short‐term ECG were significantly different from those recorded by the 24‐hr Holter‐monitoring. It may be suggested that these differences may be associated with stress‐free recording by Holter‐monitor during a long period. Finally, it can be stated that long‐term Holter‐monitoring is a more reliable technique to assess the heart electrical activities and cardiac arrhythmias of cows at different ages.

## AUTHOR CONTRIBUTION

Aliasghar Chalmeh: Conceptualization; Data curation; Formal analysis; Funding acquisition; Investigation; Methodology; Project administration; Resources; Software; Supervision; Validation; Visualization; Writing‐original draft; Writing‐review & editing. Sanaz Karamifar: Investigation; Project administration; Writing‐original draft; Writing‐review & editing.

### PEER REVIEW

The peer review history for this article is available at https://publons.com/publon/10.1002/vms3.448.
